# “Doctor, You Must Examine My Creature Collection!”: A Case Report of Delusional Infestation

**DOI:** 10.7759/cureus.25758

**Published:** 2022-06-08

**Authors:** Philip R Cohen

**Affiliations:** 1 Dermatology, University of California, Davis Medical Center, Sacramento, USA

**Keywords:** psychodermatology, psychocutaneous, parasite preservation sign, morgellons disease, ekbom syndrome, delusional parasitosis, delusional infestation, delusions of parasitosis, creature collection sign, mental health awareness

## Abstract

Delusional infestation--either secondary when attributed to a medical condition (including a bon-a-fide parasite infestation) or a pharmacologic agent (prescribed or illicit) or primary when secondary etiologies have been excluded--is a psychosis in which, for at least one month duration, the patient not only has a delusion that an animate organism or an inanimate pathogen has infested them, but also has abnormal tactile sensation (such as pruritus) of their skin caused by the etiology of their delusion. In patients over the age of 50 years, a delusional infestation is three times more common in women than men; however, delusional infestation in younger patients is often secondary, associated with illicit drug exposure, and equally common in women and men. Primary skin lesions are typically absent in delusional infestation patients; however, secondary skin lesions--resulting from the patient’s efforts to remove the parasite from their skin--can be observed, such as excoriations, prurigo nodules, scars, and ulcers. Delusional infestation patients typically strive to convince the person evaluating them that their infestation is valid and many of these individuals do this by collecting the parasites in a container. Presentation of the pathogen-filled containers is a pathognomonic feature of delusional infestation that has been referred to as either a positive creature collection sign, match box sign, parasite preservation sign, pillbox sign, or specimen sign. Morgellons disease--in which the pathogen being extruded from the skin is a fiber--has several features (including an excellent response to treatment with antipsychotic agents) in common with delusional infestation; therefore, most investigators consider Morgellons disease to be a variant of delusional infestation. Delusional infestation can be associated with numerous diseases, including comorbid psychiatric conditions. Indeed, up to 15 percent of delusional infestation patients have one (folie a deux) or more individuals with similar symptoms. A man with delusional infestation is described who had a positive creature collection sign, a fixed belief that his symptoms were caused by the infesting organism, and refusal to accept that he had a psychiatric disorder. He insisted that the evaluating dermatologist examine the pathogens in the clear plastic container he brought with him to his appointment. He was convinced that the pruritus of his scalp, eyebrows, and eyelashes was associated with a non-existent lice infestation. Secondary delusional infestation was excluded and his concurrent mild seborrheic dermatitis was treated topically; however, his itching did not resolve. He eventually agreed to seek treatment with a psychiatrist. The management of delusional infestation is based on its etiology; resolving the underlying medical condition or discontinuing the causative pharmacologic agent is the treatment approach for secondary delusional infestation, whereas low-dose antipsychotic agents are the intervention of choice for treating primary delusional infestation.

## Introduction

Delusional infestation is a monosomatic hypochondriacal psychosis. The occurrence of delusional infestation remains to be established; however, the incidence has been observed to range between 1.9 and 3.7 cases per 100,000 patients. The patient with delusional infestation has a delusion, for at least one-month duration, that they are infested with an animate organism or an inanimate pathogen. In addition, they have an abnormal tactile sensation of their skin originating from their delusion. Although the delusional infestation patient is convinced that they have an infestation, no organism is present; however, the typical features of delusional infestation may be exhibited by the patient, such as a positive creature collection sign, repeated requests for not only a thorough workup but also additional investigations to document the organism, and insistence on multiple treatments to completely eradicate the infestation [1-7].

Delusional infestation can be a primary psychosis (primary delusional infestation) or a secondary psychosis (secondary delusional infestation). Secondary delusional infestation results from either an underlying medical condition, a systemic medication, or an illicit drug; treatment is directed toward the associated etiology. Once the secondary delusional infestation is excluded, the patient is diagnosed with primary delusional infestation; for these individuals, treatment often requires antipsychotic pharmacologic intervention [7-14].

A man had a chief complaint of lice infestation-associated pruritus on his scalp, eyebrows, and eyelashes for several months duration; he had a positive creature collection sign and presented a small, clear plastic container holding several presumed organisms. A diagnosis of delusional infestation was established; eventually, the patient agreed to see a psychiatrist for definitive treatment. The features of delusional infestation are summarized.

## Case presentation

A 63-year-old man, accompanied by his wife, presented for evaluation and treatment of intractable head lice and associated itching. His past medical history was remarkable for hypertension and hyperlipidemia. He had been on daily lisinopril and atorvastatin for eight years.

His scalp pruritus had been present for three months. He had observed what he believed to be organisms; he had determined that the parasites were lice, on his scalp, his eyebrows, and his eyelashes. He shared his bed with his wife each evening; she was asymptomatic.

**Figure 1:**
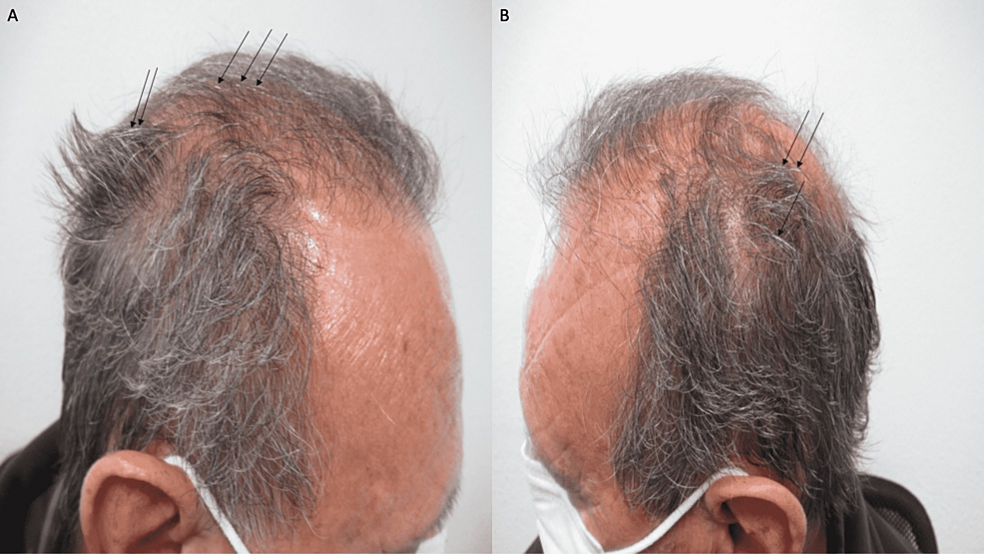
Delusional infestation presenting as scalp pruritus in a man convinced he has pediculosis The right (A) and left (B) sides of the head of a 63-year-old man, who believed that the persistent itching of his scalp for three months was caused by a lice infestation. Examination of his scalp shows androgenetic alopecia with frontal hairline recession and hair thinning on the crown and both temporal regions. Flakes of keratin (black arrows), consistent with the diagnosis of mild seborrheic dermatitis, are noted on some of the hair shafts; however, neither lice nor nits were observed.

**Figure 2:**
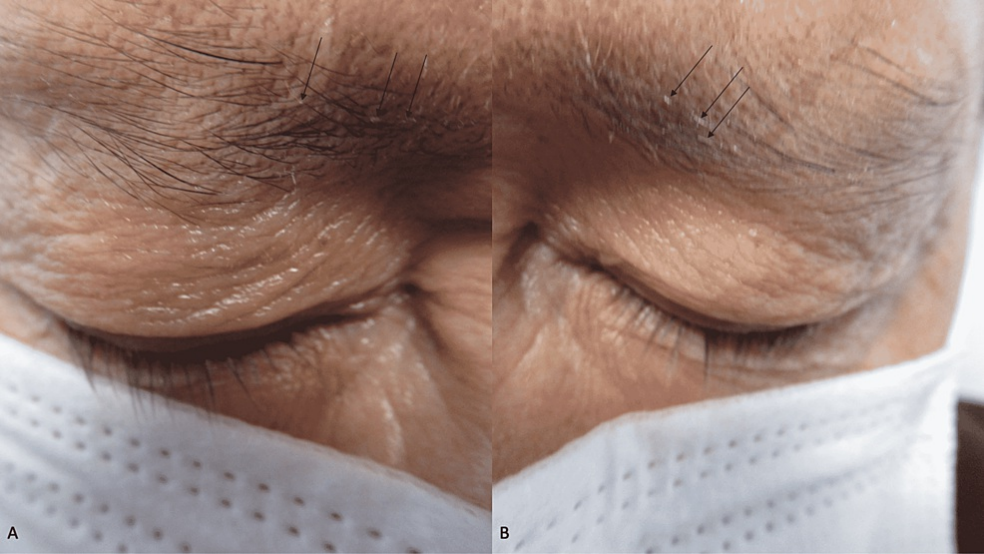
Inanimate material on the eyebrows of a man with delusional infestation interpreted by the patient to be lice The right (A) and left (B) eyebrows of a 63-year-old man show small keratin flakes (black arrows) that he is convinced are parasites.

**Figure 3:**
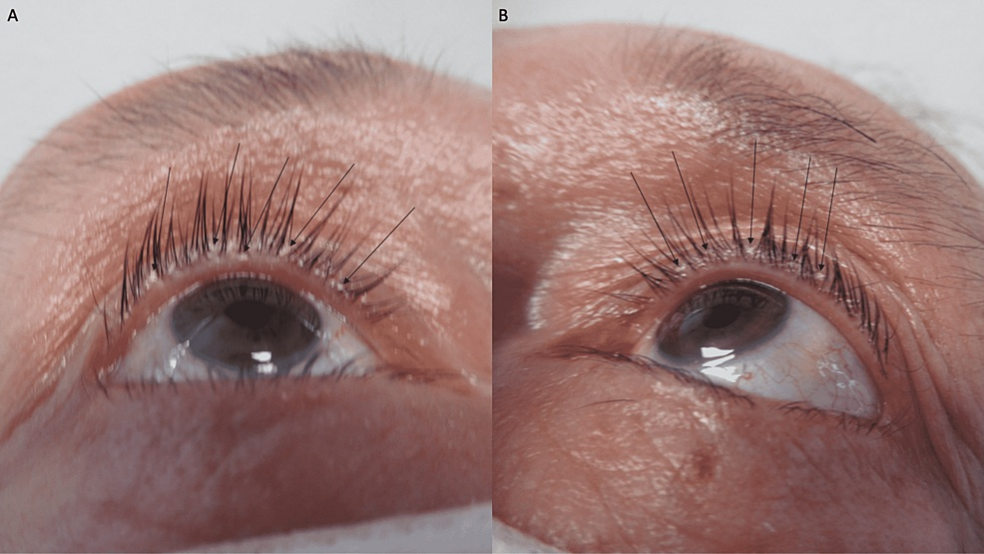
A delusional infestation patient believes that the numerous keratin plaques of mild seborrheic keratosis on his eyelashes are parasitic organisms A 63-year-old man with delusional infestation has several keratin plaques (black arrows) located at the base of his eyelashes on the right (A) and left (B) upper eyelids. He interprets the inanimate material to be lice.

A complete cutaneous examination was performed. He had male pattern alopecia demonstrated by the recession of the frontal hairline with thinning of the hair on the crown of his scalp and both temporal regions; focally, flakes of keratin were noted on occasional hair shafts (Figure 1). Similar keratin flakes were noted on both of his eyebrows (Figure 2); neither lice nor nits were observed on the scalp or eyebrow hairs. In addition, small plaques were observed at the base of both of his upper eyelashes (Figure 3).

None of his other hair-bearing areas--including his axilla and pubic regions--had similar symptoms or findings. Burrows were absent from his finger webs and there were no scrotal nodules, excluding a scabies mite infestation. His wife’s scalp and face were examined; she did not have any lice on her scalp, eyebrows, or eyelashes.

**Figure 4:**
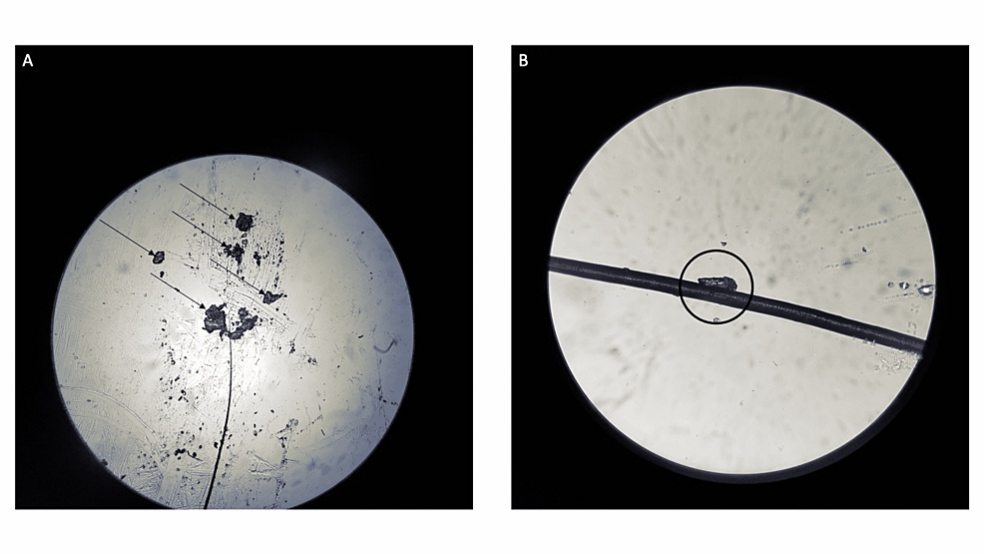
Microscopic examination of eyebrow hair and scalp hair from a man with delusional infestation Secondary delusional infestation has been excluded in a 63-year-old man with delusional infestation. He neither has a bone-a-fide parasitic infestation nor a medical condition associated with delusional infestation. In addition, he has not received any medications associated with pruritus and he has not been using illicit drugs. Hairs were obtained from his eyebrow (A) by plucking and his scalp (B) by pulling.  After placing the hairs on glass slides, they were evaluated using a light microscope. The eyebrow hair (A) has small keratin flakes (black arrows) and the scalp hair (B) has a larger keratin plaque on the hair shaft (within the black oval).

A hair was plucked from his eyebrow and another hair was pulled from his scalp. Both hairs were placed on a glass slide and examined using a light microscope. The eyebrow hair showed several small keratin flakes. The scalp hair showed a keratin plaque on the hair shaft (Figure 4).

The patient was informed that he had mild seborrheic dermatitis and that there was no evidence of pediculosis. In addition, he was told that there was no evidence of any other parasitic infestation, such as scabies. The patient then took a small, clear plastic container from his pocket and emphatically said, “Doctor, you must examine my creature collection!”

**Figure 5:**
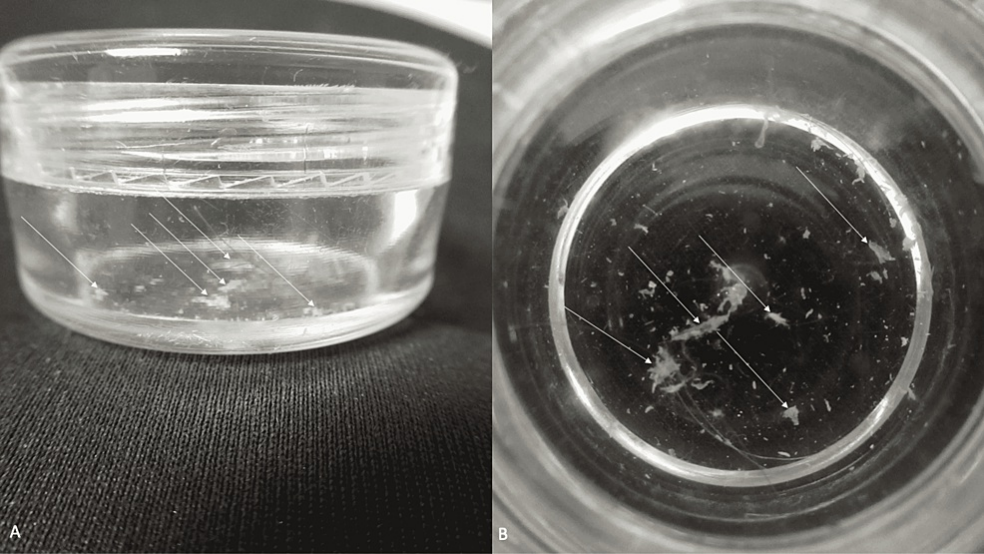
A positive creature collection sign in a man with delusional infestation A pathognomonic feature of delusional infestation is present when the patient brings their collection of what they are convinced to be pathogenic creatures for the person evaluating them to examine. The 63-year-old man with delusional infestation presented a small, clear plastic container in which he had placed what he believed to be lice that were infesting the hair on his scalp. Inanimate keratin flakes (white arrows) can be observed in the container when it is closed (A) and after it has been opened (B).

The closed container was opened and the contents were visually evaluated (Figure 5). There were numerous flakes of keratin; no lice were observed. Examination using a light microscope of pieces of the samples provided in the container confirmed the diagnosis of inanimate material. The container, which had been used to preserve what the patient was convinced were parasites, was returned to him.

Correlation of the history, clinical examination, and light microscopic evaluation of the affected hairs established the diagnoses of mild seborrheic dermatitis and delusional infestation; both diagnoses were explained to the patient. He would agree to the treatment of his seborrheic dermatitis but was convinced that lice were also present. Topical management of his mild seborrheic dermatitis, intended to provide symptomatic treatment of the pruritus, was prescribed: twice daily fluocinonide 0.05 percent solution (10 drops to his scalp and two drops on a cotton-tipped applicator applied on his eyebrows and the base of his upper eyelashes), daily washing his eyelashes with a gentle baby shampoo (using a cotton-tipped applicator), and daily shampooing of his scalp and eyebrows sequentially alternating three shampoos (which contained either 2 percent tar, 2.5 percent selenium sulfide, or 1 percent zinc pyrithione).

He returned two weeks later, accompanied by his wife. The keratin flakes and plaques on his scalp, eyebrows, and eyelashes were completely resolved. However, his scalp pruritus persisted and he again presented his container--that still only held flakes of keratin that he emphatically believed were lice--and politely insisted that I treat him for pediculosis.

The topical corticosteroid solution was discontinued and the shampooing was tapered to three times a week. His seborrheic dermatitis had cleared and no parasites were observed. However, because of his insistence, treatment--on day 1 and day 8--for pediculosis using 1 percent permethrin cream rinse that was to be applied to his scalp hair for 10 minutes and then washed off was prescribed. Also, it was suggested that he consider seeing a psychiatrist if his symptoms remained after being treated for lice; he was not receptive to this recommendation.

He returned, again accompanied by his wife, after a month. The topical treatment for lice had not been successful in relieving his symptoms. Before his follow-up visit, the results of laboratory studies that had been performed by his family doctor had been received; complete blood cell counts with platelets and a comprehensive evaluation of serum chemistries were normal. Similarly, markers of inflammation (such as C-reactive protein and erythrocyte sedimentation rate), folate and vitamin B12 levels, human immunodeficiency virus, immunoglobulin E serum level, iron and total iron-binding capacity, Lyme titers, rapid plasma reagin (evaluating for syphilis), thyroid function tests (such as thyroid-stimulating hormone, thyroxine, and triiodothyronine), and a vasculitis screen (including antinuclear antibody, antinuclear cytoplasmic antibodies, rheumatoid factor, and serologies for hepatitis A, hepatitis B, and hepatitis C) were normal or negative. In addition, his urinalysis and urine drug screen were normal and negative, respectively. Radiologic evaluation (such as a computerized tomographic scan or a magnetic resonance imaging) of his head had been suggested; however, the patient declined these studies.

In summary, there were no abnormalities in his laboratory studies. He also had not started any new systemic medications. Hence, all potential etiologies for secondary delusional infestation had been excluded. The patient was informed that there were specific agents that might ameliorate the symptoms he was experiencing. Also, it was explained to him that patients who needed these drugs may be more optimally managed by a psychiatrist familiar with the use, monitoring, and adverse effects of these medications. He once again presented his clear plastic container and was convinced that he had lice; he expressed that he wanted to continue using the shampoos and follow up again in a month.

Although his pruritus was not improving, he continued to return for monthly follow-up visits with his wife. Indeed, he had developed a rapport with his dermatologist; at each visit, a referral to a psychiatrist was offered to the patient who declined the recommendation. However, at the third follow-up visit, he commented that he would find a psychiatrist for the treatment of his symptoms and that he would only return if his itching did not improve. A year has passed and he has not returned.

## Discussion

The earliest origin of the terminology for delusional infestation is from a description by Robert Willan in 1799; he reported a patient whose condition was characterized by intense pruritus and skin scratching that was associated with a delusional belief of infestation and parasitosis. Subsequently, George Thibierge reported individuals with similar symptoms and lesions who had a delusion that their skin was infected with mites; he referred to the condition as "les acarophobes" (which has been described as either acrophobia or parasitophobic neurodermatitis) in 1894. Eventually, Karl-Axel Ekbom--a Swedish psychiatrist--described a series of eight patients who had a delusion that they were infested with the small organism in 1938; he named the condition "Praeseniler Dermatozoewahn" (which translates to presenile dermatologic delusion and has also been described as a delusion of animal life in the skin) in 1938 [3,4,7-9].

The condition was designated Ekbom syndrome; although the eponym remains, it may be confused with another disorder since the same nomenclature is used to designate restless leg syndrome. The term "delusional parasitosis" or "delusions of parasitosis" was proposed by Wilson and Miller in 1946. Other names were eventually suggested for the condition; however, they have not been frequently used in the medical literature: "chronic tactile hallucinations" by Bers and Conrad in 1954 and "monosymptomatic hypochondriasis" or "hypochondriacal psychosis" by Munro in 1988 [3,4,7-9].

In 2009, Freudenmann and Lepping proposed that the condition be called "delusional infestation." They suggested this terminology since the patients with this condition complain of infestation not only by parasites but also other small living organisms or inanimate pathogens. Subsequently, the delusional infestation has become recognized as the preferred designation for this condition [3,4,7-9].

Delusional infestation is the nomenclature that shall be used in this paper. Primary delusional infestation, a diagnosis based on the exclusion of other etiologies for the patient’s delusions, will be referred to as delusional infestation. In contrast, when the patient’s delusions of infestation are caused by either an underlying medical condition, a systemic medication, or an illicit drug, the condition shall be referred to as secondary delusional infestation [3,4,7-9].

Delusional infestation is classified in the Diagnostic and Statistical Manual of Mental Disorders (DSM-5) as a somatic type of delusional disorder. Two criteria for the diagnosis of delusional infestation have been established. First, the patient is convinced (albeit a delusion)--typically for one or more months--that they are infested by an animate organism or inanimate pathogens without any medical or microbiological evidence of a true infestation. Second, the patient has abnormal cutaneous sensations attributable to their delusion [4,7,8].

The occurrence of delusional infestation remains to be established. One study showed a prevalence is 5.6 cases per one million persons who were hospitalized or enrolled in the public health service and 83 cases per million persons in the private sector [8]. Some investigations have found the prevalence to range from 0.148 to 4.225 per 100,000 person-years and the incidence to be 0.845 per 100,000 persons [7]. However, other researchers have shown the incidence of delusional infestation to range from 1.9 to 3.7 patients per 100,000 patients [1,7].

Patients with delusional infestation present to various healthcare providers and/or non-medical personnel; this may also account for the variance of reported delusional infestation occurrence. Delusional infestation diagnoses at outpatient clinics have ranged from 0.6 patients per 1000 individuals (0.06 percent) to 20 patients per 1000 individuals (2 percent) [6]. Retrospective studies of patients admitted to the hospital for psychiatric reasons have shown the occurrence of delusional infestation to range from seven of 10,000 admissions (0.07 percent) to 67 of 1000 admissions (6.7 percent); indeed, a psychiatric clinic only evaluated 73 delusional infestation patients during a period of 29 years [5,6].

One study observed that 85 percent of dermatologists had at least one delusional infestation patient [5]. Another study of 108 dermatologists also showed that 92 (85 percent) had seen at least one delusional infestation patient. In addition, the investigation revealed that during the prior five years, 36 (33 percent) had seen one or two delusional infestation patients, and 30 (28 percent) had treated three to five delusional infestation patients [5,8].

Delusional infestation patients have also approached individuals outside of medicine regarding their condition. Pest control staff and entomologists regularly encounter delusional infestation patients [10]; consultation of patients with suspected delusional infestation by entomologists ranged from as many as 100 consultations during a period of five years (resulting in 20 consultations per year) to 77 patients during a period of 10 years (resulting in eight patients per year) [5]. In addition, a survey of 32,663 veterinarians showed that one-third of the pet owners who presented their dog or cat for evaluation or treatment of arthropod or worm infestation also claimed to be infected themselves [7].

Delusional infestation is most commonly observed in Caucasian women who are middle-aged or older. However, the delusional infestation has a bimodal distribution. The first peak is in patients between 20 and 30 years old; the second peak occurs in patients 50 years of age and older [1,5].

The ratio of women to men in the younger patients is 1:1. Secondary delusional infestation, from the use of illicit drugs, is more prevalent in younger individuals. In contrast to the younger delusional infestation patients, the ratio of women to men in the older patients is 3:1 [1,5].

Delusional infestation patients often correlated the onset of their symptoms to a specific situation. For example, it may be after either a bug bite, contact with an infected person, a sexual encounter, or sharing clothing. Alternatively, it may begin after traveling or following exposure either to someone they considered to be a dirty person or to a destination that they perceived to be a filthy place [3,4].

The duration of delusional infestation prior to diagnosis is variable; it can range from months to decades and the mean duration of disease between the onset of symptoms to the subsequent clinical diagnosis is three years. Patients with delusional infestation have often seen several individuals prior to the diagnosis of delusional infestation being established or their acceptance of the correct diagnosis, or both. Indeed, in one study, the delusional infestation patients evaluated in an emergency department had previously seen an average of six healthcare providers [3,10].

Patients with delusional infestation believe that they are infected by small living organisms. Parasites--such as lice and mites--are the most common organisms. However, some patients are convinced that the infestation is from other organisms (such as ants, fleas, insects, spiders, ticks, and worms) or pathogens (such as a bacterium, a fungus, or a virus) [1,2,7,8].

Yet, there are delusional infestation patients who believe that they are infected with non-organic, inanimate objects. These include fibers, filaments, hairs, particles, and specks. Some investigators have chosen to classify these individuals as having Morgellons disease. Indeed, in modern society, the prominent influence of the internet and social media has increased the prevalence of Morgellons disease by facilitating the interaction of individuals with others who have the same symptoms and/or condition [15-18].

Abnormal tactile sensations, such as itching, or paresthesia are the most frequent symptoms of delusional infestation; however, they also include not only burning, prickling, and tingling, but also formication--the sensation of insects crawling across and beneath the skin. Less commonly, patients with delusional infestation present with visual hallucinations; these include seeing the parasites or their eggs. Rarely, the hallucinations are either auditory (such as buzzing) and/or olfactory [1-3,7,9].

Delusional infestation patients manipulate their skin in an attempt to remove the perceived infestation. They may expose their skin to harsh cleansers and insecticides to eliminate the organisms. Also, in addition to their fingernails, they may use sharp instruments (such as knives, scissors, tweezers, and toothpicks) to extract the creatures from their skin [1-6].

Individuals with delusional infestation do not present with primary skin lesions. Their clinical manifestations of delusional infestation are more prevalent on body sites that are more readily reachable with the fingers of their dominant hand. They include secondary skin lesions, such as excoriations, prurigo nodules, scars, and ulcers; thickening of the skin (lichenification) from chronic rubbing and secondary cutaneous bacterial infections may also be observed [1-6].

The delusional infestation patient in this report had lesions of seborrheic dermatitis; yet, he did not have any delusional infestation-related cutaneous lesions. There were flakes of keratin on his scalp hair, keratin flakes on his eyebrows, and small plaques of keratin at the base of his eyelashes; he interpreted all these lesions to be lice. The seborrheic dermatitis lesions improved with topical therapy; however, the pruritus remained and his delusion of lice at these locations persisted.

A pathognomonic feature of delusional infestation has been observed in 25 percent to 75 percent of patients. It involves the patient collecting the pathogen and providing it as proof of their infestation to the person evaluating them. Indeed, several designations to describe this behavior by the delusional infestation patient have been used [1-5,7].

The name of the sign for gathering and presenting the etiologic agent responsible for the infestation emphasizes either the container used to collect the suspected pathogen or the organism. Bottles, boxes, and plastic bags have been used to collect the infesting organisms; therefore, some of the container-based names include "baggies sign," "matchbox sign," "pillbox sign," and "Ziploc sign." Alternatively, organism-based nomenclatures include "creature collection sign," "parasite preservation sign," and "specimen sign" [1-5,7].

The material collected and stored by the patient typically is composed of animal fibers, coagulated blood, cutaneous debris (such as eschars and keratin), dirt, dust, fabric, lint, plants, and/or sand. Occasionally, insect parts or a real--yet harmless--arthropod may be found; however, the patient’s symptoms or condition cannot be attributed to the organism. Indeed, only a single pubic louse was discovered in a retrospective study in which a histologic examination of 80 biopsy specimens was performed [1-5,7].

In addition to containers filled with creature collections, patients may also present digital images of the organisms that they believe are infesting them. The pictures may not only include examples--obtained by the patient--of the supposed causative parasites but also photographs demonstrating skin sites with secondary lesions where the patient has located or removed the infesting organism. Similar to the parasite preservations that may be offered to substantiate the condition by the patients, the images of the pathogens typically consist of inanimate material [1-5,7].

The patient in this report earnestly, yet with determination, requested his doctor to examine the collection of creatures he had placed in a small, clear plastic container. Using a light microscope to evaluate his creature collection only revealed keratin flakes; there were no lice or other organisms. However, even after this was explained to the patient, he still emphatically expressed that he was infested with lice.

The diagnosis of primary delusional infestation is predicated on excluding the disorder being secondary to either medical conditions or pharmacologic agents. Basic screening laboratory tests should include complete blood cell counts with platelets, C-reactive protein, erythrocyte sedimentation rate, and serum chemistries including albumin, blood urea nitrogen, calcium, creatinine, electrolytes, fasting blood sugar, liver function tests, phosphorus, and total protein. In addition, several researchers recommend additional laboratory evaluations including antinuclear antibody, antinuclear cytoplasmic antibodies, human immunodeficiency virus, iron studies (such as total iron and total iron-binding capacity), pregnancy test (in a woman of childbearing age), rheumatoid factor, serologies for hepatitis A, hepatitis B, and hepatitis C, serology for Lyme disease and syphilis (such as rapid plasma reagin), serum immunoglobulin E, thyroid function tests (such as thyroid-stimulating hormone, thyroxine, and triiodothyronine), folic acid (vitamin B9), vitamin B12 (cobalamine), and possibly other vitamin B levels including vitamin B1 (thiamine), vitamin B2 (riboflavin), vitamin B3 (niacin), vitamin B5 (pantothenic acid), vitamin B6 (pyridoxamine), and vitamin B7 (biotin), and vasculitis screening [1,5,7-9].

In addition to the initial serologic studies, a urinalysis and urine toxicology screen for drugs, such as amphetamines, benzodiazepines, cannabinoids, cocaine, and opiates (including heroin and oxycodone), should be performed. Allergy testing and a computed tomography scan of the head to evaluate for neurological lesions and/or neurological disorders have also been suggested by some investigators. Further evaluations, such as those associated with an age-appropriate cancer screen, may also be considered [1,5,7-9].

A total-body skin examination should be performed [1,5,7-9]. The detection of finger web burrows can be a clue to an unsuspected scabies infestation; light microscopic evaluation of scabies (mineral oil or potassium hydroxide) preparation of the lesion may confirm the presence of a mite, eggs, or scybala (feces) and establish the diagnosis of scabies. Similar to the patient in this report, examination of body hair for lice from the affected locations on his scalp and eyebrows using a light microscope excluded the potential diagnosis of pediculosis.

Many researchers suggest that the physician perform a skin biopsy; they also encourage that the patient selects the lesion or location to be biopsied. One group of investigators recommends not only a biopsy of a skin lesion for routine hematoxylin and eosin staining and examination but also a second, perilesional, skin biopsy for direct immunofluorescence staining and evaluation. Unfortunately, the diagnostic yield of the biopsy may be of limited value; in a retrospective study that included 80 biopsy specimens, the results--which predominantly showed chronic dermatitis, subacute dermatitis, and lichen simplex chronicus--did not alter the original clinical impression of delusional infestation [4].

The differential diagnosis of delusional infestation includes a bon-a-fide parasite infestation. In addition, dermatitis (such as those originating from agricultural grocery products, avian mites, caterpillars and moths, fiberglass, and gerbils and hamsters), dementia, pruritus secondary to systemic diseases, reactions to pharmaceutical agents (caused by either prescription medications or illicit drugs), schizophrenic spectrum disorders, and other psychiatric conditions are all possible etiologies of secondary delusional infestation. Other conditions (such as seborrheic dermatitis as demonstrated in the reported patient, dermatitis herpetiformis, the telangiectatic macularis eruptiva perstans variant of urticaria pigmentosa, and the urticarial phase of bullous pemphigoid) can also be present in patients with delusional infestation; the clinical examination can diagnose the former dermatosis whereas skin biopsy and/or appropriate laboratory studies can confirm the latter disorders [4].

Morgellons disease is a condition in which the affected patients are convinced that a material--which they describe as "fibers," "fiber-like," or "filaments"--is being extruded from their skin. Sir Thomas Browne, in 1674, described endemic distemper in the Morgellons--children of Languedoc, France--who had hairs on their back that displayed the symptoms of the disease. In 1682, the physician Michael Ettmuller produced drawings of the Morgellons fibers which were considered to be a parasitic worm infesting the children [15-18].

The term Morgellons disease was coined by a biologist, Mary Leitao, in 2002. Her son had persistent pruritus after being diagnosed with scabies and she continuously found "fibers" in his skin. Once the disease had a name, the Morgellons Research Foundation was formed; in 2002, the organization began to register patients with symptoms of the condition [15-18].

Investigators postulated an association of Morgellons disease with Lyme disease since positive Western blots for *Borrelia burgdorferi* were observed in many patients with Morgellons disease. However, the condition was not readily accepted by the medical community. In 2012, the Centers for Disease Control performed a comprehensive study of 115 self-described Morgellons disease patients in California; the results of their investigation supported a psychiatric etiology for Morgellons disease [15-18].

In summary, the affected individuals with Morgellons disease have several common features to those observed in patients with delusional infestation. Similar to delusional infestation, once non-psychiatric etiologies have been excluded, low-dose antipsychotic medications are most effective in treating Morgellons disease. Therefore, based on the considerable overlap of the disorders--especially with regard to successful management with psychiatric drug therapy--many investigators favor Morgellons disease to be a subtype of delusional infestation [15-18].

Numerous associated diseases have been observed in patients with a delusional infestation (Table 1) [1-14]. Indeed, in this setting, the disease-associated manifestations in some of the affected patients with secondary delusional infestation resolved when the primary condition was treated. In addition, some of the patients with infestation delusions have secondary delusional infestation attributable either to illicit drugs that they are using or to medications they are receiving to treat other disorders (Table 2) [1-14].

**Table 1:** Medical conditions that have been observed in patients presenting with delusional infestation Abbreviations:  AIDS, acquired immunodeficiency syndrome; HIV, human immunodeficiency virus. ^a^These include carcinoma (not otherwise specified) and neoplasia (not otherwise specified). ^b^These include chronic lymphocytic leukemia and multiple myeloma. ^c^These include hypophyseal tumor and lung cancer.

Medical condition
Autoimmune disease
Lupus erythematosus
Cerebrovascular disease
Stroke
Vascular encephalopathy
Endocrinologic disease
Diabetes mellitus
Hyperthyroidism
Hypothyroidism
Head trauma
Hematologic
Iron deficiency anemia
Hepatobiliary disease
Cholestasis
Hepatic disease
Infectious disease
AIDS/HIV
Cysticercosis
Encephalitis
Hepatitis B
Hepatitis C
Syphilis
Neoplastic disease^a^
Hematologic malignancy^b^
Solid tumor^c^
Neurologic disease
Parkinson disease
Peripheral neuropathy
Pregnancy
Psychiatric disease
Anxiety
Bipolar disorder
Delirium
Dementia
Depression
Obsessive compulsive disorder
Schizophrenia
Stress
Renal disease
Renal failure
Substance abuse disease
Alcoholism
Vitamin deficiency
Cobalamine (vitamin B12)
Folate (vitamin B9)
Niacin (vitamin B3)

**Table 2:** Illicit drugs or medications that have been observed in patients presenting with delusional infestation

Agent	Illicit drug	Medication
Alcohol withdrawal	X	
Alpha adrenergic agonists		X
Amphetamine	X	
Antibiotic		X
Ciprofloxacin		X
Antifungal		X
Ketoconazole		X
Benzodiazepines	X	
Cannabis	X	
Cocaine	X	
Corticosteroids		X
Heroin withdrawal	X	
Methadone	X	
Methamphetamine	X	
Nonsteroidal anti-inflammatory drugs		X
Opiates	X	
Parkinson disease treatments		X
Amantadine		X
Decarboxylase inhibitor		X
Entacapone		X
Levodopa		X
Levodopa/Benserazide		X
Levodopa/Carbidopa		X
Pramipexole		X
Ropinirole		X
Topiramate		X

Comorbid psychiatric diseases can be observed in patients with delusional infestation. Indeed, in one study, multiple coexisting or underlying psychiatric disorders were observed in 40 of 54 delusional infestation patients (74 percent) who were evaluated by a psychiatrist [11]. Another investigation noted that 268 of 449 delusional infestation patients (60 percent) had underlying psychiatric diagnoses [11]. The disorders included affective psychoses, anxiety disorders, depression, medical condition-induced psychoses, neurosis, obsessive compulsive disorder, organic psychosis, schizophrenia spectrum disorders, somatoform disorder, and substance-induced psychoses [1,4,7,11].

Folie a deux is another psychiatric condition that has been observed--ranging from 5 percent to 15 percent or more--in delusional infestation patients. Folie a deux, which translates to "craziness for two," is a psychiatric disorder in which two people--the patient and often a family member, relative, or close friend--share the same delusions or hallucinations. Indeed, the second person also experiences similar symptoms of delusional infestation as the patient [1,4-8,10].

This phenomenon is not restricted to just one other individual. Delusional infestation patients have had an entourage of three, four, five, or even more individuals who are similarly affected by the infestation delusion. In one situation, which was described as mass hysteria, eight workers shared delusions [9-11].

Delusional infestation by proxy has also been described in which an unaffected individual projects the delusion onto another person (such as a young child or a mentally disabled individual) or pet who cannot or does not share the delusion; the unaffected person believes that the other person or pet--but not themselves--is suffering from the infestation [7,9,11]. The third variant of delusional infestation is referred to as double delusional disorder; the patient believes that not only themselves but also another person or pet who cannot or does not share the delusion, are infested [7,9]. Recently, with the advent of widespread internet access, there are shared symptoms of specific diseases--such as delusional infestation--within focus groups; hence, folie a internet is likely to also be occurring among patients with delusional infestation [2,10].

Delusional infestation is a psychiatric condition in a patient who typically is presenting to a non-psychiatrist physician, a non-physician healthcare provider, or a non-medical person such as an entomologist or pest control employee. The patient is reluctant to accept a psychiatric diagnosis. In addition, the patient frequently will refuse a referral to a psychiatrist for additional evaluation and treatment [1,3,5,6].

However, the non-psychiatrist who has evaluated the patient and established a diagnosis of delusional infestation, after excluding other conditions and drug-associated adverse effects, may not be comfortable using the medications required to effectively treat this disorder [3-5]. Therefore, a potential alternative includes referring the delusional infestation patient to another non-psychiatrist; for example, many dermatologists refer patients with a delusional infestation to other dermatologists who often have university-based academic practices and an interest in psychodermatology. The man in this report, after many visits over several months, finally agreed to see a psychiatrist for treatment.

Once the diagnosis of delusional infestation has been established, and etiologies of secondary delusional infestation have been excluded, researchers have emphasized the importance of building a rapport with the patient [1,3,7,8]. Although psychotherapy alone has only been shown to be effective in about 10 percent of delusional infestation patients, adjuvant cognitive behavioral therapy has been observed to be useful in the management of delusional infestation [2-4,7]. However, most investigators have concluded that antipsychotic medications should be considered an essential component of delusional infestation treatment [1-4,6-8].

There are first-generation (which are also referred to as typical, such as pimozide) and second-generation (which are also referred to as atypical, such as olanzapine and risperidone), and third-generation (such as aripiprazole and quetiapine) antipsychotic medications [3]. Previously, pimozide was the treatment of choice for delusional infestation. However, this primary antipsychotic agent was associated with not only drug-related extrapyramidal effects but also the potential for cardiac arrhythmia [1-4,6-8].

Currently, risperidone--a newer, effective, and safer antipsychotic medication--is the first-line recommendation for delusional infestation; however, this drug is associated with weight gain. Second-line therapies include aripiprazole (yet there are only a few published case reports documenting its success) and olanzapine (which has also been associated with weight gain in addition to somnolence and increased lipids such as cholesterol and triglycerides). Because of the potential adverse effects associated with pimozide, this agent is now only recommended as a third-line treatment for delusional infestation [1-4,6-8].

A recent report described the successful management of delusional infestation in a 44-year-old woman using low-dose quetiapine, a second-generation antipsychotic. Her symptoms began after visiting her maternal aunt; the infestational delusion was shared by both individuals--hence, coexisting folie a deux. During a voluntary six-week hospitalization on a psychiatric ward she began quetiapine at 25 milligrams per day and increased to 50 milligrams per day; her symptoms and sleep quality improved, her distress reduced, and her tactile hallucinations resolved. She was discharged on quetiapine modified-release 100 milligrams per day [19].

All antipsychotic medications have drug-associated side effects. For secondary antipsychotics, some investigators recommend baseline laboratory studies (such as a lipid panel, fasting blood sugar, and hemoglobin A1C) at baseline and again at three months and one year. A baseline electrocardiogram is recommended if treatment with pimozide is initiated [1-4,8,20].

Remission (in between 60 percent and 100 percent) and cure with low-dose antipsychotic medication occurs in most patients with delusional infestation [1,3,4]. Indeed, the response rates of patients with delusional infestation treated with risperidone (69 percent) and olanzapine (72 percent) are similar [2,4]. Improvement is typically observed between one-and-a-half to six or eight weeks after beginning the medication [1,4,8]. Investigators recommend starting with a low dosage of the drug and gradually increasing the dosage, perhaps every four weeks, based on the patient’s clinical response to therapy [1-4,6,20]. Once a therapeutic dosage has been reached, treatment should continue for at least three months to six months after all symptoms have stopped before slowly tapering the medication [1,3,8,20].

Delusional infestation relapse has been observed in up to 25 percent of treated patients [3,4,7]. This may occur during tapering of the dosage or after the medication has been discontinued. If the symptoms recur, either the drug dosage should be increased or the medication again initiated at the dosage that previously resulted in symptom resolution [15,20].

## Conclusions

Delusional infestation is a psychiatric condition in which the patient is not only convinced that they are infested by an animate organism or an inanimate pathogen but also has abnormal tactile sensation of the skin (such as pruritus) secondary to their delusion for at least one-month duration. The diagnosis of primary delusional infestation (which is often referred to as delusional infestation) is established by excluding secondary delusional infestation which results from a medical condition or a pharmacologic agent (that has been prescribed or illicitly acquired) that can cause the same symptoms. Delusional infestation most frequently occurs in middle-aged and older women. Secondary skin lesions--such as excoriations, prurigo nodules, scars, and ulcers--may be present and result from the patient’s efforts to remove the parasite from their skin. A positive creature collection sign, matchbox sign, parasite preservation sign, or specimen sign is a pathognomonic feature of delusional infestation; the patient has collected the pathogen in a container to confirm the infestation for the person evaluating them. Typically, only inanimate material is present in the container. A man with delusional infestation and a positive creature collection sign was convinced that his scalp, eyebrow, and eyelash pruritus was associated with a non-existent lice infestation. Topical treatment of concurrent mild seborrheic dermatitis did not resolve his itching and possible etiologies of secondary delusional infestation were excluded; eventually, he agreed to seek treatment with a psychiatrist. The management of secondary delusional infestation is directed at resolving the underlying medical condition or discontinuing the causative pharmacologic agent. However, low-dose antipsychotic medication is usually an effective intervention for the treatment of primary delusional infestation.
